# Supplementary material for: MYO5A Frameshift Variant in a Miniature Dachshund with Coat Color Dilution and Neurological Defects Resembling Human Griscelli Syndrome Type 1
Source: Genes (Basel). 2021 Sep 23;12(10):1479. doi: 10.3390/genes12101479 (PMC8535926; doi:10.3390/genes12101479)
Supplement: Supplementary file 1 [file genes-12-01479-s001.zip › FigureS1.pdf]

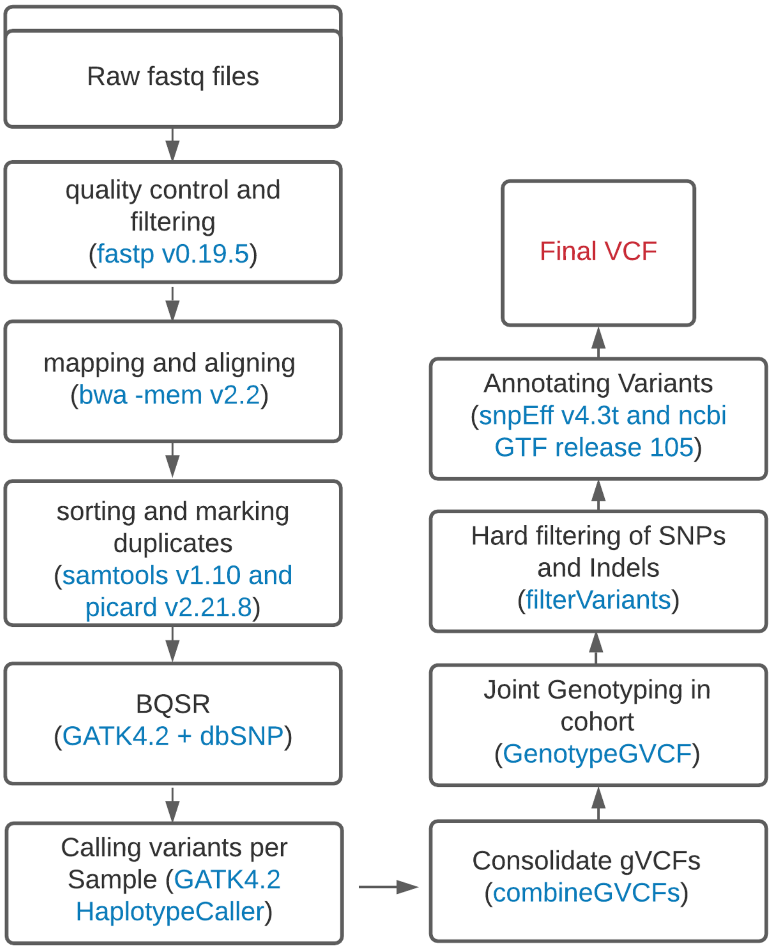

Figure S1. Mapping and variant calling pipeline. Software and modules used are shown in blue. The CanFam3.1 reference genome assembly was used for the mapping. For the base quality recalibration step (BQSR), the CanFam3.1 germline variation release 104 of Ensembl was used. The NCBI annotation release 105 for the CanFam3.1 reference genome was used for variant annotation.
